# Burden of Depression Among HIV Caregivers in India: A Systematic Review and Meta-Analysis

**DOI:** 10.7759/cureus.79697

**Published:** 2025-02-26

**Authors:** Pritam Halder, Thejas Achary, Aninda Debnath, Sayan Saha, Ashlyn Tom, Anubhav Mondal

**Affiliations:** 1 Department of Community Medicine and School of Pubic Health, Postgraduate Institute of Medical Education and Research, Chandigarh, IND; 2 Community Medicine, Vardhman Mahavir Medical College and Safdarjung Hospital, New Delhi, IND; 3 Department of Psychiatry, The National Institute of Mental Health and Neurosciences, Bengaluru, IND

**Keywords:** depression, hiv caregiver, meta-analysis, people living with hiv (plhiv), systematic review and meta-analysis

## Abstract

Caregivers of individuals living with HIV face significant mental health challenges, with depression being the most common. Despite global research highlighting this burden, studies specific to the Indian context are scarce. Addressing India’s unique sociocultural and economic factors, this review aims to estimate the prevalence and identify the correlates of depression among HIV caregivers, providing a basis for targeted interventions. Following the Preferred Reporting Items for Systematic reviews and Meta-Analyses (PRISMA) guidelines, a comprehensive search was conducted across PubMed, EMBASE, Scopus, and Web of Science. Observational studies reporting the prevalence of depression among HIV caregivers using validated screening tools were included. A random-effects model was used to estimate pooled prevalence, with heterogeneity assessed using Cochran’s Q and I² statistics. Sensitivity and meta-regression analyses were conducted to explore potential sources of variability. A total of four studies, comprising 417 caregivers from different regions of India (Bangalore, Pune, and Mangalore), were included in the meta-analysis. The prevalence of depression among caregivers varied widely across studies, ranging from 5.5% to 68.3%. Using a random-effects model, the pooled prevalence of depression was estimated at 31% (95% CI: 1-61%), indicating a substantial mental health burden among this population. Significant heterogeneity was observed (I² = 98.81%). Publication bias was assessed through Egger’s test, which indicated small-study effects, while trim-and-fill analysis did not identify any missing studies. Depression is a significant but under-researched issue among HIV caregivers in India. There is an urgent need for standardized assessment tools, routine mental health screenings, and structured psychosocial interventions to support caregivers. Future research should focus on interdisciplinary approaches and policy-driven frameworks to mitigate the caregiving burden effectively.

## Introduction and background

Caring for individuals living with HIV imposes significant emotional and psychological demands on caregivers, particularly in contexts where stigma and financial stress compound the caregiving burden. Depression, the most common psychiatric condition among caregivers, not only undermines their mental health but also compromises the quality of care they provide [1]. Globally, the intersection of caregiving and mental health has received considerable attention; however, in India, where caregiving responsibilities are predominantly informal and family-based, there remains a lack of comprehensive understanding of the mental health challenges faced by caregivers of HIV-infected individuals [2].

India, home to 2.1 million people living with HIV (PLHIV), has unique sociocultural dynamics that exacerbate caregiving challenges. Approximately 44% of HIV-infected individuals in India are in serodiscordant relationships, meaning over half a million HIV-uninfected women, primarily spouses, serve as caregivers [3]. These women face an elevated risk of depression and anxiety due to factors such as gender inequality, societal stigma, financial stress, and the constant fear of HIV transmission [4]. Studies reveal a significantly higher prevalence of psychiatric conditions among Indian caregivers of HIV patients compared to the general population [5]. These findings highlight the urgent need to address the psychological well-being of caregivers in this high-burden context.

Despite the growing recognition of caregiver mental health globally, limited research has focused on the prevalence and correlates of depression among HIV caregivers in India. Existing studies often emphasize disease transmission and treatment outcomes while neglecting the mental health of those providing care [6]. This gap is critical, as caregivers’ mental health directly influences the quality of HIV care, patient adherence to treatment, and overall health outcomes. Understanding the prevalence among caregivers can inform strategies to integrate mental health support into HIV care programs and reduce the societal burden of untreated psychiatric conditions.

This systematic review and meta-analysis aim to address these gaps by synthesizing evidence on the prevalence of depression among HIV caregivers in India. By providing a comprehensive overview of the mental health challenges faced by this vulnerable population, the study seeks to inform targeted interventions, foster mental health integration into HIV care services, and ultimately enhance the well-being of both caregivers and those they support.

## Review

Methodology

This systematic review and meta-analysis adhered to the Preferred Reporting Items for Systematic Reviews and Meta-Analysis (PRISMA) guidelines, aiming to determine the prevalence of depression among adults caring for individuals living with HIV in India [7]. The study protocol was registered with the International Prospective Register of Systematic Reviews (PROSPERO) under the registration number CRD42025633511. A comprehensive search strategy was developed to identify primary studies reporting the prevalence of depression among HIV caregivers, irrespective of the specific tools employed for depression assessment.

Study protocol and design

Observational studies, including cross-sectional, case-control, and cohort designs, were included if they reported the prevalence of depression among HIV caregivers using validated screening tools. Studies published in English up to December 31, 2024, from inception, were considered eligible. Exclusion criteria comprised studies without full-text availability, qualitative research, systematic reviews, case series, letters, and those lacking validated depression assessment tools.

Information sources and search strategy

Four electronic databases - PubMed, EMBASE, Scopus, and Web of Science-were searched up to December 31, 2024. The search strategy combined keywords and Medical Subject Headings (MeSH) terms, such as “Depression AND Caregivers OR Caring AND HIV OR Depressive Disorder AND India.” Boolean operators were employed to refine results. Supplementary Table 1 provides the detailed search strategy. Two authors (TA and PH) independently performed the searches, and the results were cross-verified to ensure comprehensive coverage. Additional sources, including reference lists and hand-searched citations, were examined to identify relevant studies not captured through database searches.

**Table 1:** Search strategy (December 31, 2024)

S. no	Database	Search Strategy	No
1	PubMed	("Parents"[MeSH Terms] OR "Single Parent"[MeSH Terms] OR "Family"[MeSH Terms] OR "Parents"[Title/Abstract] OR "Single Parent"[Title/Abstract] OR "Family"[Title/Abstract] OR "relativ*"[Title/Abstract] OR "Caregivers"[MeSH Terms] OR "caregive*"[Title/Abstract] OR "caring"[Title/Abstract]) AND ("HIV"[MeSH Terms] OR "Acquired Immunodeficiency Syndrome"[MeSH Terms] OR "HIV"[Title/Abstract] OR "Human Immunodeficiency Virus"[Title/Abstract] OR "AIDS"[Title/Abstract] OR "PLHIV"[Title/Abstract]) AND "Depression"[MeSH Terms] OR "Depressive Disorder"[MeSH Terms] OR "depressive disorder, major"[MeSH Terms] OR "Depression"[Title/Abstract] OR "Depressive Disorder"[Title/Abstract] OR "depressive disorder major"[Title/Abstract] AND "india"[MeSH Terms] OR "india"[All Fields] OR "india s"[All Fields] OR "indias"[All Fields]	52
2	Web of Science	TS=("Parents" OR "Single Parent" OR "Family" OR "relativ*" OR "Caregivers" OR "caregive*" OR "caring") AND TS=("HIV" OR "Acquired Immunodeficiency Syndrome" OR "Human Immunodeficiency Virus" OR "AIDS" OR "PLHIV") AND TS=("Depression" OR "Depressive Disorder" OR "depressive disorder major") AND TS=("India" OR "India's" OR "Indias"))	20
3	Embase	(('parents' OR 'single parent' OR 'family' OR 'relative*' OR 'caregivers' OR 'caregive*' OR 'caring') AND ('HIV' OR 'acquired immunodeficiency syndrome' OR 'human immunodeficiency virus' OR 'AIDS' OR 'PLHIV') AND ('depression' OR 'depressive disorder' OR 'major depressive disorder') AND (India OR Indias))	177
4	Scopus	(( TITLE-ABS-KEY ( "Parents" OR "Single Parent" OR "Family" OR "relativ*" OR "Caregivers" OR "caregive*" OR "caring" ) ) AND ( TITLE-ABS-KEY ( "HIV" OR "Acquired Immunodeficiency Syndrome" OR "Human Immunodeficiency Virus" OR "AIDS" OR "PLHIV" ) ) AND ( TITLE-ABS-KEY ( "Depression" OR "Depressive Disorder" OR "major depressive disorder" ) ) AND ( TITLE-ABS-KEY ( "India" OR "India's" OR "Indias" ) ))	65

Study selection

All identified citations were uploaded into Rayyan software for systematic organization and duplicate removal. Two independent reviewers (TA and PH) screened titles and abstracts to identify studies for full-text review, with disagreements resolved by discussion or consultation with a third reviewer (AD). Full-text articles were reviewed against predefined eligibility criteria, with reasons for exclusion documented at each stage. Any ambiguities regarding eligibility were resolved through consultation with the broader research team.

Data extraction

Key study characteristics - author names, publication year, study region, sample size, study design, depression assessment tools, and depression prevalence were extracted using a standardized Microsoft Excel spreadsheet. Data extraction was performed independently by two authors (TA and PH), and discrepancies were resolved through consensus.

Statistical analysis

The pooled prevalence of depression was estimated using a random-effects model to account for variability across studies. Heterogeneity was assessed using Cochran’s Q statistic and the I² statistic. Subgroup analyses explored variations based on gender. Sensitivity analyses, leave-one-out analyses, were conducted to identify studies disproportionately contributing to heterogeneity and to test the robustness of pooled estimates. Funnel plots and Egger’s regression test were used to assess publication bias. Trim-and-fill analysis was applied to adjust for potential bias by imputing missing studies. Meta-regression analyses evaluated the influence of covariates such as sample size and mean age of the participants on the pooled prevalence, with results visualized through bubble plots. Statistical significance was set at p < 0.05, and all analyses were conducted using STATA 18 (StataCorp. Stata statistical software: Release 18. College Station, TX: StataCorp LLC; 2023).

Quality assessment and risk of bias

Two independent reviewers (TA and PH) assessed the methodological quality of the included studies using the Joanna Briggs Institute (JBI) Critical Appraisal tools for prevalence studies [8]. Studies were categorized as poor (scores 1-4), fair (scores 5-6), or good (scores 7-9). Higher scores indicated a lower risk of bias, ensuring that the findings were based on methodologically robust studies.

Results

**Figure 1:**
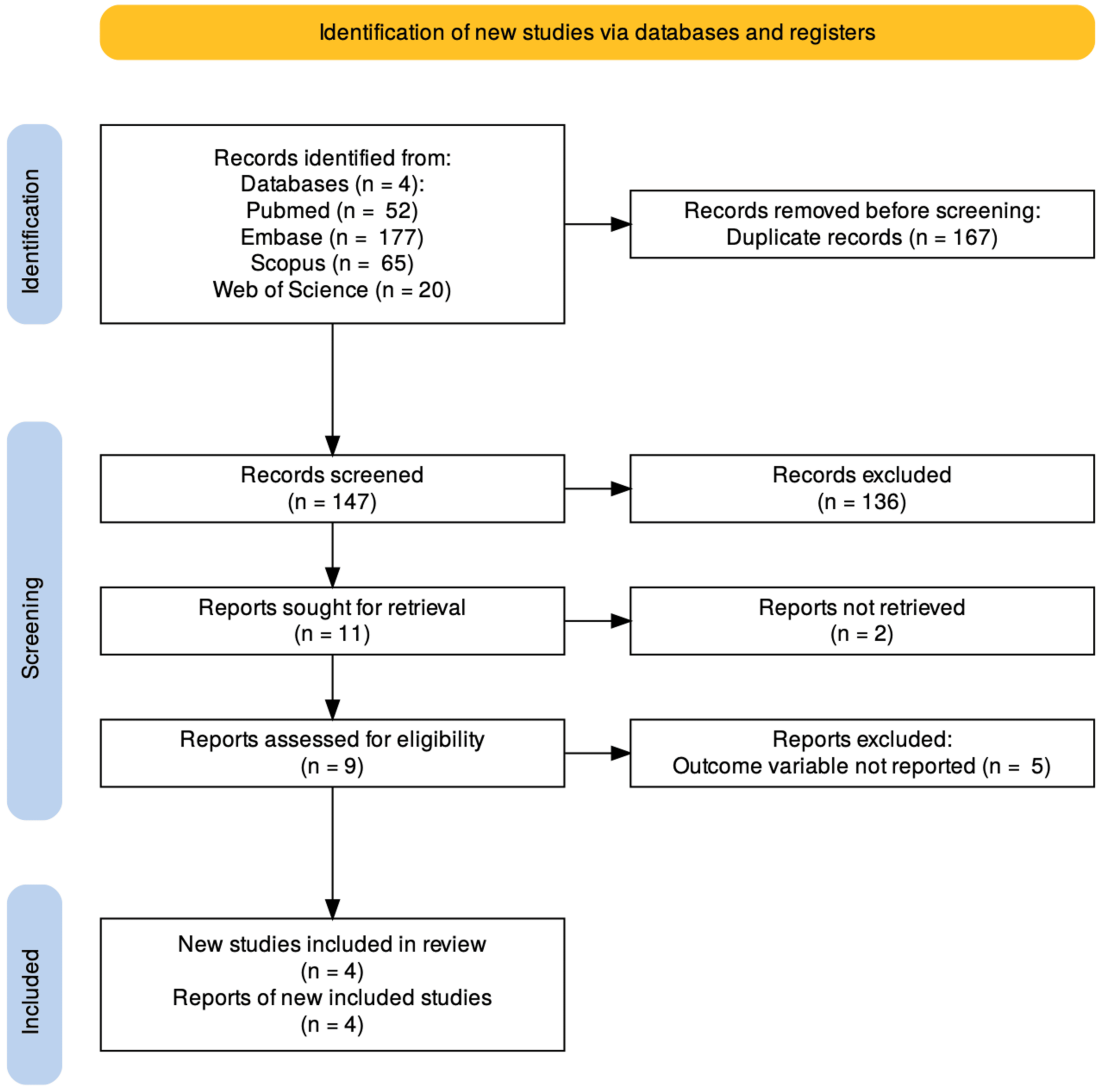
PRISMA flow diagram PRISMA: Preferred Reporting Items for Systematic Reviews and Meta-Analysis The image was created by the authors of this article.

The systematic search across four electronic databases yielded 314 records. After removing 167 duplicate entries, 147 unique records were screened for relevance based on titles and abstracts. Of these, 136 records were excluded for not meeting the inclusion criteria, leaving 11 reports for further assessment. Full texts were retrieved for two studies, with five excluded due to the lack of reporting on the primary outcome variable. Ultimately, four studies were included in the systematic review and meta-analysis, as detailed in the PRISMA flowchart (Figure 1).

A total of four studies were included in this meta-analysis, conducted in different regions of India, including Bangalore, Pune, and Mangalore. Three of the studies were hospital-based, while one was community-based. The sample sizes ranged from 55 to 152 participants, with a total of 417 participants across all studies. Depression was assessed using various validated tools, including the Structured Clinical Interview (DSM-IV), Beck’s Depression Inventory, Depression Anxiety Stress Scales (DASS-21), and the Mini International Neuropsychiatric Interview (MINI). The prevalence of depression among caregivers varied significantly across studies, ranging from 5.5% to 68.3% (Table 2). In the quality assessment of the study, all the studies were of good quality (Table 3).

**Table 2:** Characteristics of the studies included for meta-analysis

S. No	Author (Year of Publication)	Study Site	Sample Size	Mean Age of the Participants	Tools to Assess Depression	Number of People Suffering From Depression	Prevalence Of Depression
1	Pandit and Vishnuvardhan (2014) [9]	Bangalore	60	31.2	Structured clinical interview (DSM-IV)	41	68.3%
2	Ghate et al. (2015) [10]	Pune	55	34.27	Beck’s Depression Inventory	3	5.5%
3	Khan et al. (2018) [11]	Mangalore	150	39.6	Depression Anxiety Stress Scales	69	46%
4	Darak et al. (2019) [12]	Pune	152	40.1	Mini International Neuropsychiatric Interview	10	6.6%

**Table 3:** Quality assessment of the studies included in the meta-analysis Q1 Was the sample frame appropriate to address the target population? Q2 Were study participants sampled in an appropriate way? Q3 Was the sample size adequate? Q4 Were the study subjects and the setting described in detail? Q5 Was the data analysis conducted with sufficient coverage of the identified sample? Q6 Were valid methods used for the identification of the condition? Q7 Was the condition measured in a standard, reliable way for all participants? Q8 Was there appropriate statistical analysis? Q9 Was the response rate adequate, and if not, was the low response rate managed appropriately?

S. no	Author (Year of publication)	Q1	Q2	Q3	Q4	Q5	Q6	Q7	Q8	Q9	Score	Quality
1	Pandit and Vishnuvardhan (2014) [9]	Yes	Yes	No	Yes	Yes	Yes	Yes	Yes	Yes	8/9	Good
2	Ghate et al. (2015) [10]	Yes	Yes	Yes	Yes	Yes	Yes	Yes	Yes	No	8/9	Good
3	Khan et al. (2018) [11]	Yes	Yes	Yes	Yes	Yes	Yes	Yes	Yes	Yes	9/9	Good
4	Darak et al. (2019) [12]	Yes	Yes	No	Yes	Yes	Yes	Yes	Yes	No	7/9	Good

**Figure 2:**
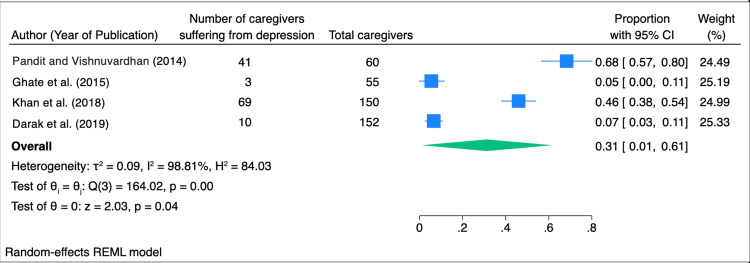
Forest plot of depression prevalence among HIV caregivers included in the meta-analysis Pandit and Vishnuvardhan [9], Ghate et al. [10], Khan et al. [11], Darak et al. [12]. The image was created by the authors of this article.

A total of four studies involving 417 caregivers of individuals living with HIV were included in this meta-analysis, of whom 123 were identified as suffering from depression. Using a random-effects model, the overall pooled prevalence of depression among caregivers was estimated to be 31% (95% CI: 1-61%). Substantial heterogeneity was observed across the studies (I² = 98.81%, τ² = 0.09, p < 0.01), indicating significant variability between studies. Cochran’s Q test confirmed the presence of heterogeneity (Q = 164.02, p < 0.01) (Figure 2).

**Figure 3:**
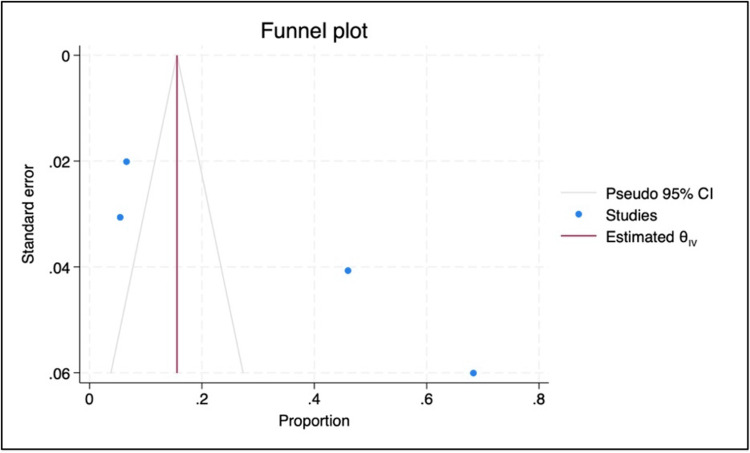
Funnel plot showing publication bias The image was created by the authors of this article.

Publication bias was assessed using funnel plot symmetry, Egger’s regression test, and trim-and-fill analysis. The funnel plot showed moderate asymmetry. Egger’s test further confirmed the presence of small-study effects, yielding a significant result (β = 17.15, p < 0.001). However, the trim-and-fill analysis did not identify any missing studies, and the pooled proportion remained unchanged at 31% (95% CI: 1-61%) (Figure 3).

**Figure 4:**
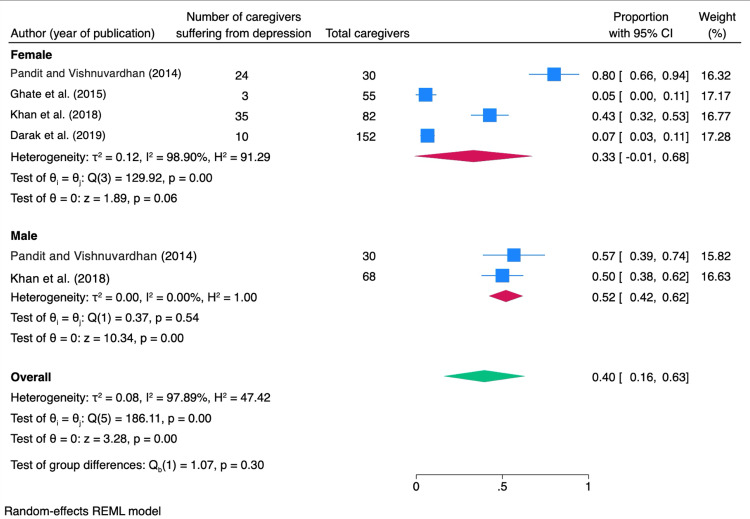
Forest plot showing subgroup analysis of the pooled prevalence of depression among caregivers of PLHIV, stratified by gender PLHIV: people living with HIV Pandit and Vishnuvardhan [9], Ghate et al. [10], Khan et al. [11], Darak et al. [12]. The image was created by the authors of this article.

The subgroup analysis stratified by gender revealed a pooled prevalence of depression among females of 33% (95% CI: 1%-68%) with substantial heterogeneity (I² = 98.9%), while for males, the pooled prevalence was 52% (95% CI: 42%-62%) with lesser heterogeneity (I² = 0%). A test for group differences between males and females showed no statistically significant difference (Q = 1.07, p = 0.32) (Figure 4).

**Figure 5:**
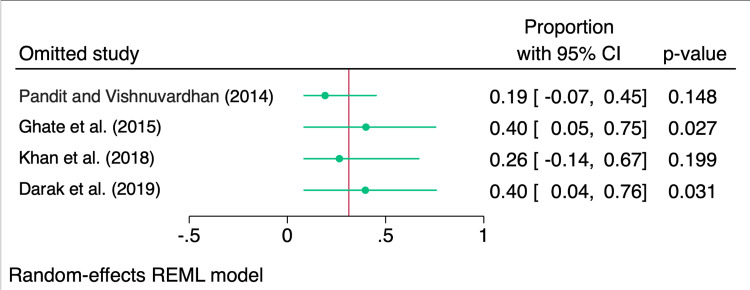
Leave-one-out analysis showing the impact of omitting each study on the pooled prevalence Pandit and Vishnuvardhan [9], Ghate et al. [10], Khan et al. [11], Darak et al. [12]. The image was created by the authors of this article.

A leave-one-out sensitivity analysis was performed to assess the robustness of the pooled proportion by systematically excluding each study and recalculating the results. The analysis showed that omitting certain studies, particularly Ghate et al. (2015) and Darak et al. (2019), had a significant impact on the pooled proportion, with proportions recalculated as 0.40 (95% CI: 0.05, 0.75) and 0.40 (95% CI: 0.04, 0.76), respectively, both with significant p-values. In contrast, excluding Pandit et al. (2013) and Khan et al. (2017) resulted in non-significant changes to the pooled estimate, with proportions of 0.19 (95% CI: -0.07, 0.45) and 0.26 (95% CI: -0.14, 0.67) (Figure 5).

Discussion

This systematic review and meta-analysis, which synthesized data from four studies encompassing 417 caregivers of individuals living with HIV across various regions in India, estimated the pooled prevalence of depression among caregivers of individuals living with HIV in India at 31% (95% CI: 1-61%).

Studies globally have consistently reported a higher prevalence of depression among caregivers of individuals living with HIV compared to the general population. Research from diverse regions, including Nepal, Africa, and South Asia, underscores the psychological toll of caregiving, with depression rates ranging between 28% and 65%, depending on contextual factors such as gender, financial constraints, and caregiver burden. In Nepal, nearly 45% of caregivers experienced mild to severe depression, with caregiver burden positively correlating with depression levels [13]. Similar trends have been observed across African nations, where depression prevalence among caregivers of PLHIV ranged from 13.4% to 64.5%, influenced by factors such as low income, social stigma, and female gender [14,15]. Notably, in rural Uganda, 61% of female caregivers exhibited clinical depression and anxiety, while studies from Kenya and Nigeria reported high depression scores on validated scales [15,16].

The results highlight a significantly higher prevalence of depression among caregivers of PLHIV compared to national estimates from the National Mental Health Survey (NMHS) 2015-16 and the Global Burden of Disease (GBD) 2017 study [17,18]. This prevalence also surpasses that observed among caregivers of cancer patients, a group that has been extensively studied in terms of psychological distress [19]. The stress proliferation model offers a critical lens to understand these findings, emphasizing how the financial, physical, and psychological burdens of prolonged caregiving take a significant toll on mental health [20]. Beyond the direct challenges of caregiving, other psychosocial stressors - such as guilt among biological parents, social isolation, limited disease knowledge, difficulties with disclosure, and HIV-related stigma - further exacerbate depression in this population [21].

HIV-positive caregivers face heightened psychological distress, given their dual burden of managing their own chronic illness while simultaneously providing care. This distress is compounded by financial difficulties, treatment costs, and service shortages, all of which pose substantial barriers to their well-being [22]. A qualitative study on the impact of HIV caregiving on family systems in India identified pervasive themes such as stigma and discrimination, challenges in disclosure, changes in family dynamics, financial insecurity, and overwhelming feelings of helplessness, all of which contribute to poor mental health outcomes [23].

Other factors linked to higher depression rates among informal caregivers include coexisting HIV and non-HIV medical conditions, substance use, absence of additional caregiving support, extended time spent with the patient, and the duration since the patient’s diagnosis [6]. These findings indicate that depression among caregivers is not merely an individual psychological response but rather an outcome of systemic stressors that require structured intervention strategies [24,25]. The high prevalence of depression in this population aligns with findings from prior studies, further justifying the need for targeted support mechanisms.

The expansion of community-based HIV care has placed increased reliance on informal caregivers, particularly in low- and middle-income countries (LMICs) like India, where resources are limited and family members play a central role in care. However, caregivers remain underrecognized in healthcare systems, often viewed as a dispensable resource rather than key stakeholders requiring structured support. For sustainable, high-quality HIV care, it is imperative to integrate caregiver well-being into broader HIV care frameworks. To address these challenges, structured interventions must be implemented at both the policy and healthcare levels. Routine depression screening programs at antiretroviral therapy (ART) units should be established to ensure early detection and intervention [13]. Additionally, there is a pressing need to develop psychosocial interventions targeting emotional stability, family relationships, parent-child dynamics, coping mechanisms, and problem-solving strategies in conjunction with pharmacological management, where necessary, to alleviate caregiver distress [26,27]. Healthcare professionals should also be trained to identify and address caregiver distress, incorporating self-care strategies into their interactions with families.

The mental health burden, including depression, among HIV caregivers remains an underexplored area in the Indian context. There is a critical need for more primary research to comprehensively assess the extent of this issue. Future studies should prioritize evaluating the effectiveness of proposed interventions while also investigating innovative, interdisciplinary strategies to alleviate caregiver distress. Given that caregiving presents multifaceted challenges - medical, social, and economic - at the household level, addressing these complexities requires a comprehensive policy framework that goes beyond individual psychological interventions to encompass systemic support mechanisms.

This study adheres to PRISMA guidelines, ensuring transparency and methodological rigor. A high level of heterogeneity (I² = 98.81%) was observed in our meta-analysis, reflecting substantial variability among the included studies. This heterogeneity can be attributed to differences in study populations (hospital-based vs. community-based), depression assessment tools (DSM-IV, Beck’s Depression Inventory, DASS-21, MINI), and sociocultural factors across study locations. To account for this, we employed a random-effects model, which assumes variation in effect sizes across studies. However, despite these adjustments, significant residual heterogeneity remained, suggesting that depression prevalence among HIV caregivers is influenced by multiple contextual factors. Future research should incorporate stratified analyses based on caregiver roles, gender, and regional disparities to better understand these variations.

By integrating robust statistical methods, including random-effects modeling, subgroup analyses, and sensitivity testing, our study provides comprehensive insights into the prevalence of depression among HIV caregivers in India. However, wide confidence intervals, particularly in subgroup analyses (e.g., females: 33% (95% CI: 1%-68%)), indicate substantial uncertainty in prevalence estimates. This variability is likely due to small sample sizes within individual studies and demographic differences among participants. While our pooled prevalence estimate offers a general indication of the mental health burden among caregivers, the wide confidence intervals underscore the need for larger, more representative studies with standardized depression assessment tools to improve the precision of future findings.

A major limitation of this meta-analysis is the small number of included studies (n = 4) with a total sample size of 417 caregivers. This limited dataset restricts the generalizability of our findings and may have contributed to both high heterogeneity and wide confidence intervals. While our analysis provides valuable preliminary insights, future research should prioritize multi-center studies with larger and more diverse caregiver populations to enhance statistical power and improve the reliability of prevalence estimates.

## Conclusions

This systematic review and meta-analysis highlight the substantial burden of depression among caregivers of individuals living with HIV in India, with an estimated pooled prevalence of 31%. The findings emphasize the urgent need to integrate mental health support into HIV care frameworks, particularly by addressing systemic stressors such as stigma, financial hardship, and gender disparities. Caregivers play a critical role in HIV care, yet their well-being remains largely overlooked in healthcare planning and policy. Recognizing caregivers as an integral part of the healthcare system and prioritizing their mental health is essential for sustaining high-quality HIV care and improving health outcomes for both caregivers and patients. Addressing these challenges through structured, evidence-based interventions is not only a public health necessity but also an ethical imperative, ensuring sustainable and equitable healthcare for those providing and receiving care.
